# Meta‐audit of laboratory ISO accreditation inspections: measuring the old emperor's clothes

**DOI:** 10.1002/mbo3.314

**Published:** 2015-12-01

**Authors:** Ian G. Wilson, Michael Smye, Ian J. C. Wallace

**Affiliations:** ^1^ Northern Ireland Public Health LaboratoryBelfast City HospitalCorry Building, 51 Lisburn RoadBelfastBT9 7ABUnited Kingdom; ^2^ Biochemistry LaboratoryRoyal Victoria HospitalKelvin Building, Grosvenor RoadBelfastBT12 6BAUnited Kingdom; ^3^ Tissue Pathology and Molecular LaboratoryBelfast City HospitalGardner Robb Building, 51 Lisburn RoadBT9 7ABUnited Kingdom

**Keywords:** Accreditation, audit, ISO 17025, microbiology, public health, United Kingdom Accreditation Service (UKAS)

## Abstract

Accreditation to ISO/IEC 17025 is required for EC official food control and veterinary laboratories by Regulation (EC) No. 882/2004. Measurements in hospital laboratories and clinics are increasingly accredited to ISO/IEC 15189. Both of these management standards arose from command and control military standards for factory inspection during World War II. They rely on auditing of compliance and have not been validated internally as assessment bodies require of those they accredit. Neither have they been validated to criteria outside their own ideology such as the Cochrane principles of evidence‐based medicine which might establish whether any benefit exceeds their cost. We undertook a retrospective meta‐audit over 14 years of internal and external laboratory audits that checked compliance with ISO 17025 in a public health laboratory. Most noncompliances arose solely from clauses in the standard and would not affect users. No effect was likely from 91% of these. Fewer than 1% of noncompliances were likely to have consequences for the validity of results or quality of service. The ISO system of compliance auditing has the performance characteristics of a poor screening test. It adds substantially to costs and generates more noise (false positives) than informative signal. Ethical use of resources indicates that management standards should not be used unless proven to deliver the efficacy, effectiveness, and value required of modern healthcare interventions.

## Introduction

A dominant theme in clinical science since the 1990s has been the rise of evidence‐based medicine (EBM). EBM is not universally agreed upon but sits in contrast to command and control micromanagement and governance by measures which are inappropriate, wasteful, and counterproductive. Descriptions of such operations in the literature include “audit society” (Lawrence 2007) and “probophilia” (Kenny and Davies 2015). A publish‐or‐perish culture fueled by bibliometric assessment is the familiar manifestation of probophilia in academia. In analytical laboratories, a major form of inappropriate measures is accreditation to ISO management standards. Contradictions between EBM and accreditation exist because there is very limited evidence (Shaw et al. 2014) that ISO management accreditation achieves what it claims and criticisms have not been addressed adequately.

It remains unproven whether accreditation does any good (Anonymous, 2011). Outcomes have not been compared to establish whether accreditation changes quantitative results or quality of service that is noticed by users. Further investigation is essential because the expenditure on accreditation is large. Thorough evaluation would be required before any similarly expensive pharmaceutical intervention would be adopted.

### Legislative background

Accreditation is an inspection system that checks compliance with proprietary (closed) ISO standards through manuals of Standards Operating Procedures (SOPs), exhaustive records, and multiple layers of verification. It has described these as a *quality* or *management system*. The United Kingdom Accreditation Service (UKAS) (Wikipedia, 2015a) is the assessment body with the monopoly in the United Kingdom to conduct accreditation. It is part of an international network of measurement, inspection, and standards organizations that have many of the characteristics of a cartel. Regulation (EC) No. 882/2004 (European Commission, 2015) requires a network of National Reference Laboratories and Official Control Laboratories in member states. It mandates that these must be accredited to the laboratory management standard ISO/IEC 17025 and includes agricultural, veterinary, public analyst, and public health laboratories.

### Historical development

The laboratory management standards ISO/IEC 17025 and ISO/IEC 15189 were derived from management standards including BS 5750 and the ISO 9000 series. Since this is not known by many scientists the background needs to be considered in some detail. ISO/IEC 17025 recognized ISO/IEC 9001 as inadequate to assure the validity of analytical results:

Conformity of the quality management system within which the laboratory operates to the requirements of ISO 9001 does not of itself demonstrate the competence of the laboratory to produce technically valid data and results. (ISO/IEC 17025:2005, 2005).

The clauses of ISO/IEC 17025 go far beyond the requirement of ISO 9001 to have a management system that can be inspected in order to assure that laboratories “operate a management system, are technically competent, and are able to generate technically valid results” (ISO/IEC 17025:2005, 2005). It replaced ISO/IEC Guide 25, EN 45001, and UKAS M10 and M11.

The management standards are vague enough to be sold widely and therefore must employ supplementary technical standards in specific laboratory settings. They originated in command and control (Seddon 2005) inspection systems dating back to World War II Defense Standards that relied on factory inspectors (Seddon 2000b) and were derived from the American National Standards Institute (ANSI) standard. The historical controversy about the scientific management movement and critiques of management standards (Marshall 2006) is rarely discussed in laboratories by assessors or in the scientific literature, however major problems are clear in fields such as the food industry (Townsend and Gephardt 1995; Powell et al. 2013), engineering (O'Connor 1998; Williams 2012; Heffner et al. 2015), software engineering (Weir 2010), business management and occupational psychology (Seddon 2000a, 2004a; Raventos 2011), public sector services (Guilfoyle 2012; Langford 2014), chemistry (Anonymous, 2006), medicine (Petersen 2003; Wilson 2013; Kenny and Davies 2015), and the corruption and failure of ISO 9000‐inspired quality assurance in universities (Charlton and Andras 2002; Stone and Starkey 2011). These references should be consulted since it is beyond the scope of this paper to evaluate in detail the recurring criticisms across a broad range of expertise. ISO standards may offer valid choices for products that are mass manufactured but their effects, at best, remain to be proven for the management of human behavior.

### Ontology

The nature of quality is an ancient and complex debate between whether quality is intrinsic to an object or attributed by the observer. The ISO definition of quality has been described as “almost impenetrable to those not familiar with the world of standards” (Burnett 2002) and to “have evolved over a number of years” (Burnett 2013). By defining quality as the “degree to which a set of inherent characteristics fulfills requirements” the ISO covers both aspects and is not inconsistent with the popular definition used for most decisions: *fitness for purpose*. However, the principles of harmonization and subsidiarity (ISO/IEC 17025:2005, 2005; Burnett 2013) have been invoked to give an appearance of standardization and to commercialize quality assessment for third parties. This has enabled a shift of decision making from users to professional assessors and world governance organizations (Murphy and Yates 2009) so that the ISO definition of quality is, in practice, *the ability to be inspected for compliance*. It is the assurance of a bureaucratic process as a metaphor for quality, which in itself can indicate neither high nor low quality (Anonymous, 2009a; Heffner et al. 2015).

Accreditation is the checking of compliance with the local implementation of proprietary international standards. Assessment through auditing is the procedure by which this is done and is the essence of accreditation. However, auditing is an analogy drawn from accounting practice where financial error and fraud may be detected *in closed systems* where accounts should balance. Therefore audit can only address performance synecdochically, presuming the parts that are inspected represent the entirety of an operation. It is unsuitable to be relied upon without validation *in open systems* where the process rather than the outcome is checked (Charlton and Andras 2002).

Assessors must remind staff at every visit that their assessment is only a sampling exercise. Despite the implication of technically valid results that are the basis for international assurance (ISO/IEC 17025:2005, 2005), a warranty of perfect fitness for purpose clearly cannot be made without total control of every element at all times. This could carry legal liability. Since the sampling possible during an annual assessment by the external body is very limited, the credibility of the assessment methodology is superficially strengthened by a requirement for frequent internal audits of compliance with the numerous clauses of the ISO standards and technical supplements. Assessment bodies therefore can sell “confidence” to governments and customers while evading the legal responsibility one would expect from an assurance that was valid.

### Absence of critical evaluation

Accounting practices in our setting do not allow accreditation costs to be distinguished from other expenditure, but we estimate that servicing the demands of accreditation has increased costs by around a third. Despite years of accreditation, no data are available on the numbers needed to treat (NNT) or to what degree accreditation meets the Cochrane requirements of efficacy, effectiveness, and value for ethical healthcare interventions (Järvinen et al. 2011). The inspection industry has persuaded governments that accreditation is exceptional but its *prima facie* plausibility is not sufficient to exclude it from fuller accountability. Accreditation is an extravagant and demanding process. It relies entirely on other industries that have long provided satisfactory services without accreditation and it should not be exempted from the standards applied to the pharmaceutical industry. International accreditation bodies assess each other to ISO/IEC 17020:2012. Since they share the ISO philosophy and hold monopolies in their own countries, this exercise depends on circular reasoning. It does not prove assessment works; validation must come from a higher scientific standard outside the inspection movement itself.

Intriguingly, the design of the ISO management standards may preclude external validation to the standards required for other clinical treatments. Therefore, we investigated accreditation by combining its own principle of auditing compliance with an independent, semiquantitative evaluation of the risks and effects of noncompliances on the validity of test results and quality of service to users.

## Methods

Several internal audits were carried out in a regional public health laboratory each month by laboratory staff between 2000 and 2013. The standards used in 2000 were NAMAS M10 and M11. After 2001 the standard was ISO/IEC 17025:2000 which was revised in 2005. Further analysis of the audits was not planned as they were being done. In a retrospective meta‐audit of these audits, two clinical scientists, experienced in accreditation but independent of the laboratory, reviewed the audit records and reassessed the original findings. They evaluated the potential effect of each noncompliance with the ISO standard on the validity of results and quality of service through application of their own knowledge, questioning, and discussion. To ensure a correct understanding of the noncompliances in their laboratory setting and to minimize bias, one scientist from the audited laboratory was available to respond to their questions but did not take a directive role in the reassessment.

ISO/IEC 17025 relies on an idiosyncratic definition of quality using a binary compliance/noncompliance decision by a single assessor. The objectivity of this is unproven. The design of this process effectively elevates the unimportant to the imperative and compels wasteful activity on issues that contribute no value (Seddon 2005). The meta‐audit therefore reassessed each audit decision with a second level of categories that used risk evaluation and related to the normal definition of quality as *fitness for purpose* rather than *compliance*. The criteria used to reassess each finding were *the likelihood and severity of invalid results or poor service quality*. These were categorized as *unlikely* (a noncompliance with ISO requirements only and that had no clear consequence for results or service), *possible* (technically capable of contributing to an invalid result or poor service and reasonably likely to do so in this setting at some point), *likely* (an invalid result or clearly poor service would probably occur).

To control for auditor bias in internal laboratory audits, a selection of external audits conducted annually against ISO/IEC 17025:2005 by a similar number of different UKAS assessors was also subjected to meta‐audit. In this case, the categories of no noncompliances and internal failure reports (IFRs) and complaints were excluded because these categories were null. For both internal and external audits the original audit findings were those of a single auditor. The categories recorded in the meta‐audit were the agreed opinion of two assessors.

## Results and Discussion

### Effects of noncompliances on the validity of results and quality of service

**Figure 1:**
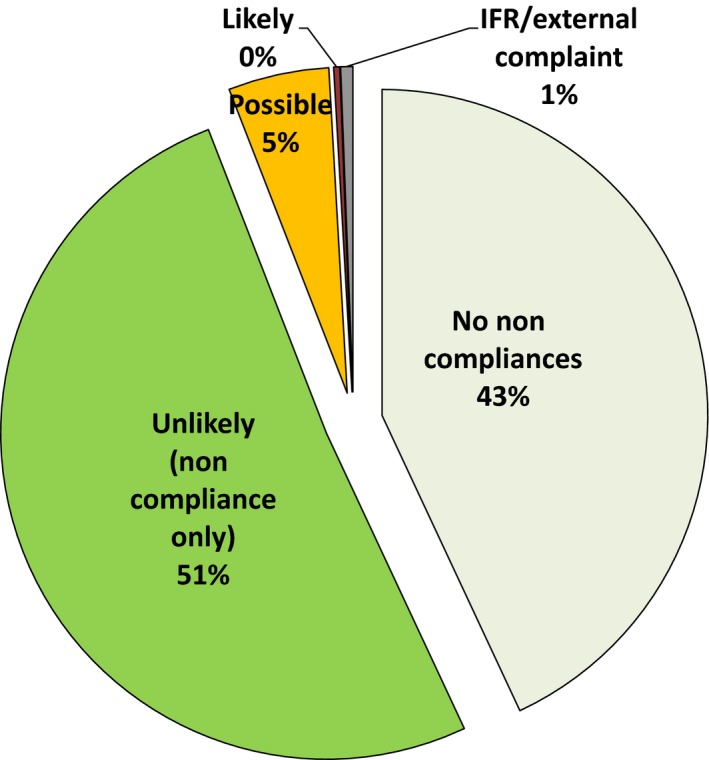
Audit findings and effects of noncompliances on validity of results or quality of service.

Three hundred and thirty‐three audits conducted between 2000 and 2013 detected 188 noncompliances. The audit findings and effects of noncompliances on the validity of results or quality of service are shown in Figure 1. No noncompliances with ISO/IEC 17025 were found in 143 (43%) of audits, and 170 audits (51%) showed noncompliances that related purely to the ISO standard. Their nature indicated that there were no likely effects on results or service quality. Seventeen audits (5%) showed noncompliances that in some circumstances may possibly have compromised results or service at some time. Two audits (0.6%) were undertaken because of prior items discovered outside the audit process through issues reported internally or externally. They were not a measure of the ability of audits to detect issues that would otherwise go unrecognized, merely investigations of their causes.

One audit (0.3%) revealed a single noncompliance would have been likely to have affected results. This was considered likely to have had a deleterious effect, although, on subsequent investigation, its significance was debatable since only EQA samples were affected. Procedural errors with a computerized colony counter gave incorrect aerobic colony counts in defined circumstances. The errors affected only five EQA plates because these are more frequently overgrown with *Bacillus* spp. than routine plates. Although considered in both internal and external audits, the error was not discovered through them. It was detected as a result of five EQA samples which exceeded the acceptable limits of the expected value over 10 months but without a positive or negative bias. The root causes were investigated and corrected.

The conclusion of this meta‐audit was that almost half of audits revealed no noncompliances. In over half, the issues were noncompliance with clauses of the ISO standard only, not with failings likely to influence the validity of results or quality of customer service. It would therefore be more accurate to say that the requirement for compliance with ISO 17025 was the root cause of apparent problems rather than the solution for real and significant quality failures.

### Consequences of noncompliances

**Figure 2:**
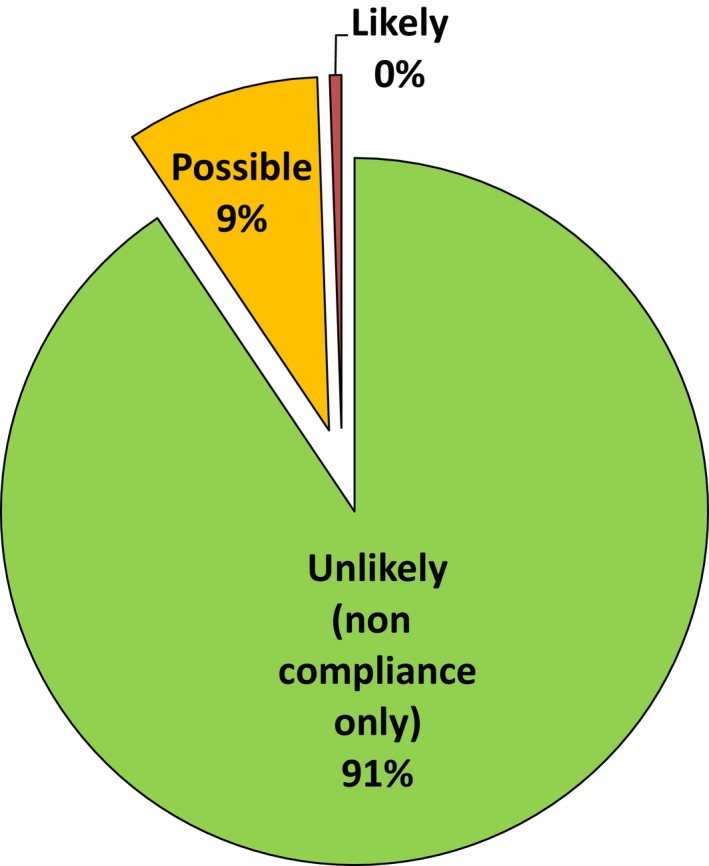
Effects of noncompliances on validity of results or quality of service.

Figure 2 shows an assessment of the likely consequences of the noncompliances discovered in audits. The scale is more informative than Figure 1 since it excluded audits which detected no noncompliances or were initiated because of failures discovered outside compliance audits.

In this retrospective study without denominator data it was not possible to calculate performance characteristics for the processes of accreditation such as true and false positives and negatives, sensitivity, specificity, likelihood ratio, positive and negative predictive values, and numbers of tests or laboratories needed to treat to evaluate the performance of accreditation and whether it has any value. Nevertheless, the meta‐audit data allow an estimation of some characteristics by analogy. Figure 1 and 2 illustrate the true positive and true negative rates. They show the weak statistical performance of compliance audits as a binary classification function and indicate that accreditation leads to substantial misdirected effort and waste.

Solving known quality problems is not the reason accreditation is sought by laboratories. It is used to provide assurance of inspection for those with no apparent problems and is therefore a screening instrument. Auditing for compliance is analogous to a patient screening test with low sensitivity for issues important to the validity of results and quality of service. It is a characteristic of screening tests that the proportion of false negatives may be high when the condition is common. Correspondingly, the proportion of false positives tends to be high when the prevalence of the condition is low. Some auditors will detect very minor differences between written procedures and actual practice but without assessing their significance realistically beyond the small proportion of samples that can be audited. Auditing therefore tends to detect many minor details because of the requirements for documentary and procedural minutiae to be correct. This boosts the detection rate of issues unimportant to genuine quality which should be classified as false positives.

Perhaps accreditation, if used as a diagnostic test, could improve laboratories that are “unwell” but it is wasteful and unhelpful as a screening test for the routine discovery of critical issues in laboratories which are clearly “healthy” in terms of good IQC and EQA performance. This is because accreditation was conceived merely as a management tool without an understanding of clinical testing. Assessment may mask other issues by focusing attention on the irrelevant noncompliances (false positives) that have no influence on genuine quality. Important failures are more likely to be detected more promptly by observant staff, IQC or EQA than by compliance audit.

Such performance characteristics are widely understood to indicate a poor screening test that is inadequate for clinical service. Its use may be counterproductive. Many medical screening tests give rise to unnecessary concern, harmful investigations and abuses, and are frequently unhelpful (Krogsbøll et al. 2012; Haelle 2013; Teirstein 2015). A “conspiracy of silence” about their deficiencies has been observed (Colquhoun 2014a). These meta‐audit data are consistent with the ISO standard having been written to justify inspections that maximize the number of improvement actions. This gives a consistent, but misleading, impression of value rather than focusing on the issues of importance. It is incongruent that laboratory staff members fail to apply to accreditation the performance criteria they use to assess laboratory tests.

The ISO management standards provide a pretext for alleging poor quality and apparently proving it through subjective interpretations on points that are usually unimportant. Guilt rather than innocence is the presumption and is used to justify exhaustive record keeping as a verification ritual. Inspection acts as a nocebo, creating unjustified doubt and false emergencies. From this follows the placebo of reaccreditation which, it is claimed ( http://www.ukas.com), provides the emotion of “confidence.” The strategy mirrors the use of ISO standards as international trade barriers and accreditation as the *de facto* tariff that opens markets (Townsend and Gephardt 1995).

This form of inspection has ethical implications because accreditation is perpetual treatment for an undiagnosed condition and offers no prospect of a cure since extensive investigation of the Scottish Quality Management System (SQMS) found that “tangible benefits did not accrue” when ISO 9000 “was unilaterally imposed on a dependant population to a degree whereby around a third of the population relied on achieving SQMS in order to survive and therefore had no choice in the matter” (Marshall 2006). Marshall (2006) concluded that, This research therefore corroborates many of the research findings for ISO 9000 and makes a very important contribution to the argument that the compulsory imposition of a Management Standard is likely to be counter‐productive. The much more stringent laboratory management standards have the potential to be more counterproductive than ISO 9000 and ethical use of healthcare resources requires that the value of accreditation must be evaluated (Perneger 2004) to establish whether it is noninferior to having no accreditation.

### Nature of noncompliances

**Figure 3:**
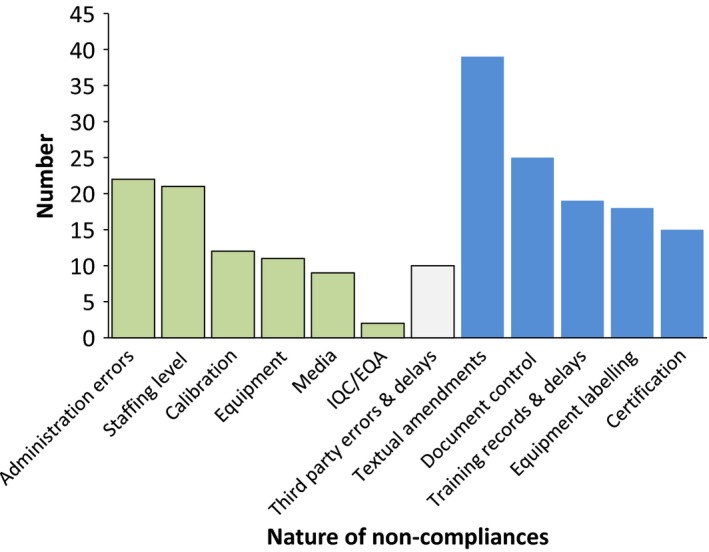
Nature of noncompliances.

Figure 3 shows the distribution of noncompliances by their nature. Green bars show areas that arose because of laboratory issues. Blue bars show issues created solely by the ISO standard. Third‐party issues sometimes arose from accredited or certificated companies and were outside the control of the laboratory. Noncompliances that made invalid results possible or likely were mostly distributed in the first 2 years when staff members were inexperienced with accreditation. These related to media production, adequate staffing, training records, and resource problems.

The nature of noncompliances that arose due to laboratory issues included administrative errors, inadequate staffing, equipment, media, IQC, and EQA failures. The majority of noncompliances related to minor textual amendments to SOPs and control of document versions. A small number of errors and delays arose from third parties, mostly ISO‐certified or accredited equipment companies. Sticking identification labels on equipment, keeping extremely complex training records up to date, and pursuing ISO certificates from suppliers were common noncompliances that arose from ISO 17025 alone.

### Sources of noncompliances

**Figure 4:**
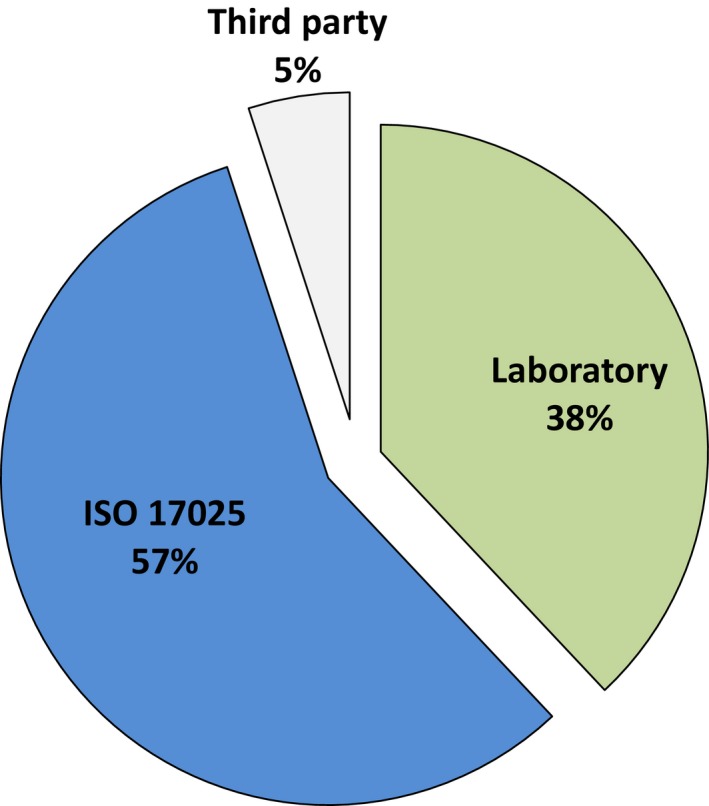
Sources of noncompliances.

Figure 4 used the same dataset as Figure 3 and shows how the sources of noncompliances arose from the laboratory, third parties, or the ISO standard's requirements alone. Laboratory failings were responsible for 38% and third parties for 5%. The requirements of ISO 17025 alone were the root causes of 57%. This distribution indicates that compliance audits generate more noise than signal. They are a poor use of resources because their effects on the quality of results and service are minimal.

The majority of noncompliances are unimportant for anything but demonstrating attempts to comply. Almost twice as many noncompliances related to equipment not having identification labels than to equipment being defective. The correctness that accreditation assures is the process of inspecting samples of processes, not assuring genuinely valid results. “Confidence” and “assurance” are claimed to arise from knowing that compliance inspection is carried out regularly even though accreditation does not guarantee error‐free reporting when it describes results as “technically valid.” These emotions are ironically nonmetrical for a body whose origins lie in calibration and may have been chosen as a result of earlier controversies and problems with unsupportable advertising claims and alleged corruption (Stone and Starkey 2011; Anonymous, 2006; Seddon 2004b). It has not been shown how compliance inspection is an advance on assuring quality by other means and the argument for accreditation is defective. Recognizing this limitation has not kept accreditation from forming a central part in the future planned for pathology (Barnes 2014). In other industries, some are turning from the waste of compliance inspection to systems that improve quality and efficiency.

### Importance of noncompliances and consistency between internal and external auditors

**Figure 5:**
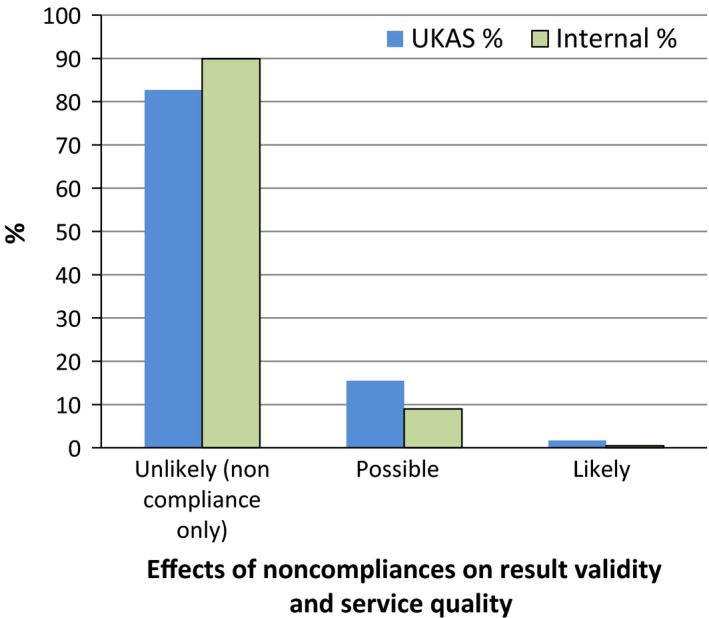
Comparison of United Kingdom Accreditation Service and internal auditors on the likelihood and severity of invalid results or poor service quality.

Four annual audits conducted by pairs of six different UKAS lead and technical assessors were sampled as controls for bias or deficiencies in the skills of internal laboratory auditors and detected 58 noncompliances. Figure 5 shows that broadly similar proportions of noncompliances were found between internal and external auditors when their findings were categorized according to the likelihood and severity of an important effect arising. Internal auditors tended to raise noncompliances over clerical issues. External auditors were inclined to raise noncompliances over adverse events they speculated might occur. Noncompliances genuinely likely to result in invalid results or poor service were extremely rare with either set of auditors. Since the role of accreditation in delivering real quality was negligible, laboratories should stop the waste of supporting redundant assessment bodies and instead prioritize appropriate and efficient testing for customers.

### Sources of uncertainty

The semi‐objective data of noncompliances were transformed into quantitative data to enable enumeration and analysis. This gave rise to uncertainty from minor discrepancies that arose for reasons such as whether to count the continuance or recurrence of a noncompliance in a follow‐up audit, closely related noncompliances having been counted together in one original audit but separately in the other, and noncompliances that related to more than one category for analysis or whose nature was ambiguous. These affected a minor proportion of the total noncompliances assessed and would have had a very limited effect on the shape of the distributions since similar events were usually in the same categories.

No complaints were received, so audit findings could not be related to poor service perceived by customers. Occasional suboptimal and incorrect EQA outcomes occurred. These generally arose from equipment and human factors that were not controlled by the accreditation requirements and included data entry, dilution, and mixing errors.

Evaluation of the significance of findings in this meta‐audit was limited by the same subjectivity as external assessors. However, it was strengthened by being the agreed opinion of two independent evaluators who were not under managerial pressure to find noncompliances as a measure of their effectiveness. The design of future meta‐audits should plan for improved control of such uncertainties. Now that the distribution of effects is apparent, prospective design of audit records for subsequent meta‐audit could eliminate most of these uncertainties by including an appropriate impact assessment during the initial audit. Strict definitions for the anticipated effect could reduce variations in opinion between auditors.

The findings apply to audits of compliance with management standards and probably do not apply where audits are performed outside the accreditation system for the purposes of understanding and improving operations. ISO management standards have subsumed quality practices such as IQC, EQA, and the Deming wheel for continual improvement. But accreditation is not needed for these to have value.

### Evidence and the assessment of accreditation

#### Scope and strength of evidence

It has been increasingly understood that failure to publish negative results distorts the evidence base on which decisions are made (Altman and Moher 2013; Colquhoun 2014a). Critical scientific reports of applying ISO management accreditation to laboratories are surprisingly few. Most papers are descriptive and lack objective evidence that accreditation has brought value or even improvement. Their strength of evidence is equivalent to a single‐center case report which is a weak basis for clinical decision making. Many publications fail to rise above the lowest level of evidence, level 5 (Expert opinion without explicit critical appraisal, or based on physiology, bench research or “first principles,” Anonymous, 2009b). Higher quality studies are rare, indirect, and much less supportive. Reports that are critical mostly relate to the ISO 9000 series which is widely established in nonlaboratory industries. They are often in blogs and books rather than the scientific literature, although the authors frequently have experience of accreditation in a number of workplaces. For this reason most scientists have remained unaware of critical evaluations outside PubMed and the general lack of evidence to support the accreditation industry.

#### Proficiency testing evidence

The data available are very limited but at the other end of the scale from single‐center descriptive reports, proficiency testing schemes have the potential to give insight into the long‐term effectiveness of accreditation. Despite the metrical perfectionism of assessors, a study funded by the UK Food Standards Agency (FSA) reported that the reproducibility of microbial counts in routine enforcement examination of foods averaged ±12% and ranged up to ±41% (Jarvis et al. 2007). Results from a national proficiency testing program of over 39,500 samples in the United States, where ISO accreditation is not common, provide an imperfect control but an informative comparison in which over 5% of food pathogens failed to be detected (Snabes et al. 2013). A similar magnitude of pathogen detection failures occurred in a proficiency testing program which serves mainly accredited UK and European laboratories.

Disclaimer: The opinions are those of the authors.

[Correction added on 21 December 2015, after first online publication: Nita Patel and Public Health England have been removed from the above sentence and a disclaimer has been added to this current version.] These percentages varied over time and between pathogens and appear consistent with Gaussian distributions and overlapping confidence intervals. Detailed analysis that might identify notable international methodological variations or causal association with accreditation has not been published. Both enumeration and detection figures would not surprise microbiologists of the preaccreditation era. They appear unsupportive of the carefully nonspecific claims of accreditation to transform the validity of results within or between laboratories. Therefore publication of proficiency testing data is needed to determine whether ISO accreditation has any effect on the results that laboratories report. If accreditation lacks the power to affect numerical results and service quality to users, its alleged value is negated.

#### Confounding factors

Publications may be confounded by factors including lack of objectivity, various biases and fallacies, the source of commissioning or funding, and the large numbers that now derive their income from writing, explaining, and administering the requirements of the assessment system (O'Connor 1998; Seddon 2004a). Variations in the performance of a service may arise from correlating factors such as the attention to detail inspection stimulates or contemporaneous, multifactorial organizational changes, rather than from accreditation itself (Walshe 2009). Paradoxical effects that diminish quality have not been excluded (Øvretveit and Gustafson 2003). Appropriately designed trials would be required to clarify causality, as for other expensive healthcare interventions.

A large proportion of science is in error (Ioannidis 2005; Colquhoun 2014b) and obsessive compliance inspection is misdirected in attempting to correct this. Negligible evidence has been offered to support the implication that accreditation satisfactorily controls all factors that might affect results. It may be possible to show if inspection has some value as a transitional tool in laboratories that are very poorly managed but further research is needed to determine the extent to which accreditation has any value generally (Brook 2010).

There are ethical, philosophical, practical, and economic reasons to question the application of a treatment where the efficacy, effectiveness, and value of a healthcare intervention cannot be demonstrated. The presumption of guilt rather than innocence is at odds with an established legal tradition that can restrain autocracy. Senior assessment managers do not intervene in the judgments of lead assessors, so reasoning against arbitrary opinions requires a cumbersome, two‐stage appeal process. Most laboratories are not aware of this option. The presumption is a double standard because while assessors accept nothing as true without documentary evidence that can be inspected, customers are compelled to multiply layers of evidence for inspection and to believe in the process of assessment without evidence that it works. The increased workload serves to prevent reflection and investigation. Accreditation's surprising endurance relies on political influence and marketplace coercion (Seddon 2000a; Marshall 2006; Wikipedia, 2015b) through discrimination against suppliers who are not accredited or certified. This is a characteristic of a cartel and is of questionable morality and legality. One reason for its toleration is how accreditation fits into a wider administrative ideology (Seddon 2014). A second double standard is revealed by our experience from investigating work done inadequately by suppliers that ISO 17025 is applied much less stringently to commercial companies than to publically funded bodies.

The assertions and limited data available have the appearance of having been fitted to conform to the dogma of inspection and more objective evidence is needed to establish that the claims for accreditation are not bogus. As accreditation spreads through all areas of clinical measurement, the results of this meta‐audit will be informative across a wide range of scientific, medical, and engineering disciplines.

## Conclusions

We have provided evidence that compliance audits according to the ISO 17025 standard are, at best, very inefficient tools to assure quality. Almost all noncompliances were inconsequential. They feature constantly in every accredited laboratory and are exonerated by recurring assessment and accreditation without notable adverse or beneficial effects. These results are consistent with the ISO standard having been written to give an impression of a legitimate process rather than to correct noteworthy problems. The requirement for compliance with ISO 17025 was much more often the root cause of issues than the solution for significant quality failures.

This investigation should encourage others to develop meta‐auditing and other studies for the assessment of laboratory accreditation by proven standards outside its own philosophy. Meta‐audit in other laboratories will enable the gap to be evaluated between accreditation according to its own ideology (“a collection of ideas that are used to advance or maintain the authority and power of their exponents in a way that prevents critical analysis of whether the ideas are true or false, consistent or inconsistent.” [Hayes 2009]) and objective standards designed for ethical critical assessments, without a vested interest.

We believe this analysis clearly distinguishes the difference between meaningful quality, as commonly understood, and the inspection process marketed as “quality.” We anticipate that a broadly similar pattern will be seen across the scientific disciplines into which accreditation spreads. It should be understood as one of a succession of management fads (Abrahamson 1996; Walshe 2009) and actions without evidence (Doern and Onderdonk 2014). Buist and Middleton (2013) summarized the situation:

Despite the hundreds of millions of dollars that have been consumed by the quality and safety industry…nothing much has changed.

A paradigm shift rejecting high‐cost, low‐value bodies with a similar business model is beginning (Teirstein 2015). Laboratory professionals, doctors, managers, and politicians should examine the cost, effectiveness, ethics, and legitimacy of accreditation. They should stop the magical thinking that it requires and consider how superior quality could be delivered by simpler and more efficient arrangements.

Lord Kelvin remarked, “Anything that exists, exists in some quantity and can therefore be measured.” Accreditation without measurable performance characteristics is not science but a bureaucratic ritual involving the inspection of science; homeopathy for laboratories. It is as surprising as it is ironic that no disinterested party has offered a scientifically valid measure of performance for an inspection organization whose original role was assuring calibrations. To assess the assessors, we should build on the data in this report, remember *The Emperor's New Clothes* and seriously question whether any genuine quality is brought into existence by this form of inspection at all.

[Correction added on 11 December 2015 after first online publication: The Acknowledgement Section has been deleted and Note has been added on page 1.]

## Conflict of Interest

I.J.C.W. is a quality manager. I.G.W.'s duties include quality manager.
